# Associations of canopy leaf traits with SNP markers in durum wheat (*Triticum turgidum* L. *durum* (Desf.))

**DOI:** 10.1371/journal.pone.0206226

**Published:** 2018-10-23

**Authors:** Sisi Huang, Longqing Sun, Xin Hu, Yanhong Wang, Yujuan Zhang, Eviatar Nevo, Junhua Peng, Dongfa Sun

**Affiliations:** 1 College of Plant Science and Technology, Huazhong Agricultural University, Wuhan, Hubei, China; 2 Institute of Evolution, University of Haifa, Mount Carmel, Haifa, Israel; 3 College of Agriculture, Guangdong Ocean University, Zhanjiang, Guangdong, China; 4 Life Science and Technology Center, China National Seed Group Co., Ltd, Wuhan, Hubei, China; 5 Hubei Collaborative Innovation Center for Grain Industry, Changjiang University, Jingzhou, Hubei, China; Institute of Genetics and Developmental Biology Chinese Academy of Sciences, CHINA

## Abstract

The canopy leaves including the top three, i.e., the flag, the 2nd and 3rd from the top, are important for photosynthesis and grain yield of wheat. Molecular markers associated with traits of these leaves should be helpful for the high-yielding breeding. In this study, 1366 single nucleotide polymorphisms (SNP) markers covering the whole genome of durum wheat were used to genotype 150 cultivars collected from 46 countries and regions in the world. Leaf length, leaf width and chlorophyll content of the top three leaves were measured, respectively, in three consecutive years. Association analyses were performed on the leaf traits and SNP markers. A total of 120 SNP marker associations were detected on 13 of the 14 chromosomes. Among these markers, 83 were associated with the canopy leaf traits, 10 with 1000-grain weight, and 29 with kernel number per spike. This study is helpful for better understanding the potential and genetic basis of functional leaves, and facilitates pyramiding of the favorable alleles using marker assisted selection for ideal plant-type and high photosynthesis efficiency in durum wheat breeding.

## Introduction

Wheat (*Triticum* spp.) is one of the major food crops that widely planted in the world [1]. Plant type, leaf type and spike type are the three important factors impacting crop yield [2]. Through photosynthesis plants use sunlight energy to convert water and carbon dioxide into organic matter/carbohydrate, the basic resources of crop yield formation. Previous studies showed that 95% of cereal crop yield were derived from photosynthesis [3]. Leaf is the major plant organ for photosynthesis in wheat. The organic matter and energy of photosynthesis can be stored in the leaf photosynthetic organelles and transferred into other parts of the wheat plants [4]. Among the 12–13 leaves of the whole plants, the canopy leaves including the top three leaves (the flag leaf, the 2^nd^ and the 3^rd^ leaf from the top) are the most important for the entire life cycle of wheat [5,6], and produce more than 80% primary nutrients accumulating in the wheat grain by photosynthesis after heading [7]. It has been reported that the photosynthesis efficiency of wheat leaves is not only influenced by the leaf morphological traits, such as leaf length, width and area, but also affected by the chlorophyll content [8,9]. Thus, the morphology and chlorophyll content of leaf are the main factors impacting conversion of sunlight to sugars in plant [10]. Enhancing photosynthesis efficiency is really an important approach of improving grain yield. Thus leaf traits are among the key objectives in wheat breeding.

The accumulation of dry matter in wheat shoot mostly come from photosynthesis, and the flag leaf account for 45% - 58% of the total photosynthesis products of all leaves [11]. Previous studies have shown that flag leaf provides 41% to 43% of the carbohydrates for grain filling [12]. Therefore, flag leaf is one of the main sources of wheat grain carbohydrates. However, the morphological traits of flag leaves, such as length, width and area, directly affect their photosynthesis efficiency. Thence, it is of great significance to study genetics of the flag leaf morphological traits for improving wheat grain yield.

At present, many QTL have been identified for the morphological traits of flag leaves in rice and barley [13,14], while few are reported for wheat flag leaves. Keller et al. [15] found eight QTL controlling leaf width at chromosomes 1A, 1B, 2A, 3B, 5A, 5B and 6A. Jia et al. [16] mapped three major QTL controlling flags on 1B, 3A, and 4A, and three major QTL conferring leaf margin at 2D and 5A (*QFlw*.*nau-2D*, *QFlw*.*nau-5A*.*1* and *QFlw*.*nau-5A*.*2*). Xue et al. [17] mapped the gene *TaFLW1* for flag leaf width into the Xzmw482—Xzmw752 interval of 0.2 cM on chromosome 5A. Eleven QTL controlling flag leaf width were detected and mapped to chromosomes 1B, 2A, 2B, 3A, 4D, 5A, 6B and 7D by Wu et al. [18]. At the same time, they mapped the QTL for flag leaf length into 7 chromosome intervals with PVE = 3.48% - 23.86%, and QTL for flag leaf area into 13 chromosome regions with PVE = 3.33% - 26.13% [18]. Liu et al. [19] detected three major QTLs on chromosome 2D, 4D and 5B for the flag leaf length in common wheat. Using available simple sequence repeat linkage map, Liu et al. identified 23 QTLs for FLL, FLW, FLA and FLANG on chromosome 1B, 2B, 3A, 3D, 4B, 5A, 6B, 7B and 7D in wheat [20].

The regulation mechanism of chlorophyll content is very complicated. Any variation related to chloroplast differentiation and chlorophyll metabolism can lead to the change of chlorophyll content, and leaf color variation manifested is shown. In addition, some of the genes that indirectly regulate chlorophyll metabolism and the pathway of chloroplast differentiation and development may lead to the change of chlorophyll content. In the model organisms such as Chlamydomonas, Arabidopsis and rice, all enzymes involved in chlorophyll biosynthesis have been identified [21]. However, due to the large size and complexity of wheat genome, only a few studies about the chlorophyll biosynthesis were conducted. Recently, a new incomplete dominant yellow-green gene *Y1718* in common wheat was identified and mapped to chromosome 2BS using molecular markers [22].

Based on the linkage disequilibrium (LD) of alleles, association mapping analysis can be performed to reveal relationship between molecular markers and target traits [23–27]. So far, association analysis has been widely used in many important crops, such as barley, maize, soybean, etc. [28–32]. However, due to the complexity of the wheat genome, association mapping studies in wheat lags far behind diploid crops, such as rice, maize and barley. With development of the high-throughput DNA sequencing technology, single nucleotide polymorphism (SNP) markers would make association analysis more efficient and cost-effective [33–35].

Cultivated wheat consists of mainly two species, the hexaploid bread wheat (*T*. *aestivum*) and tetraploid durum wheat (*T*. *durum*) [36]. The modern breeding technology and cultivation practices lead to loss of a large number of beneficial alleles and narrow genetic basis in common wheat. The breeding process has not only resulted in weak resistance or tolerance to biotic and abiotic stresses, but also seriously restricted the efficiency of trait improvement in wheat [37,38]. It is of great significance to broaden the genetic base of common wheat by exploring and bringing in excellent genetic resources from the relative species. Durum wheat (AABB) and its wild relative, wild emmer wheat, carry more abundant beneficial alleles for yield and quality traits than bread wheat, and can serve as a natural gene bank for common wheat improvement [36]. In this study, we performed association analysis on the targeted canopy leaf traits in a set of durum wheat germplasm collected globally. The results can be helpful for fine mapping and cloning of genes conferring leaf traits and for molecular marker-assisted selection in wheat.

## Materials and methods

### Plant materials and field trials

One hundred and fifty durum wheat germplasm accessions collected from 46 countries and regions in the world were used in the study [35,39]. During the 2014/2015, 2015/2016 and 2016/2017 cropping seasons, the durum wheat accessions were planted in late October on the experimental farm of Huazhong Agricultural University, Wuhan, China. All of the materials used in this study were provided by Dr. Junhua Peng requested from USDA (United States Department of Agriculture) and no any protected species were sampled in the field trials. The experimental field belongs to the type of heavy loam with PH value of about 6.2. Each accession was sown in four rows with 1 m in length, 0.2 m between rows, eight hills in each row, field management is consistent with common wheat conventional field management. The randomized complete block design was adopted with three replications. The wheat plants were supported by bamboo sticks to prevent lodging after heading.

### Trait measurement

At flowering stage, 6 plants with uniform vegetative and reproductive growth, and without disease and pests were randomly chosen from each accession. The leaf length, width and chlorophyll content of the canopy leaves, flag leaf, the 2^nd^ and the 3^rd^ leaf form the top were measured, respectively. Mean of the 6 plants was calculated as the phenotypic value for the specific leaf traits of a genotype.

The leaf length was measured as the distance from the leaf ear to the leaf tip. The leaf width referred to width at the widest part of the leaf. The leaf area was calculated as length × width × 0.75, as described previously [40]. Chlorophyll content of the three upper leaves was determined by SPAD-502 Chlorophyll Meter (Model SPAD-502 and KONICA MINOLTA, INC. JAPAN), at the top, middle, and bottom part of every leaf, respectively. The mean of three measurements from the three spots was calculated as the chlorophyll content of each measured leaf. The phenotypic data of kernel number per spike were collected in the year 2010 to 2013 by Hu et al. [35].

In total, 16 traits were measured or calculated: FLL, the flag leaf length (cm); FLW, flag leaf width (cm); FLA, the flag leaf area (cm^2^); FLCC, the flag leaf chlorophyll content; SLL, the upper second leaf length (cm); SLW, the upper second leaf width (cm); SLA, the upper second leaf area (cm^2^); SLCC, the upper second leaf chlorophyll content; TLL, the upper third leaf length (cm);TLW, the upper third leaf width (cm); TLA, the upper third flag leaf area (cm^2^);TLCC, the upper third leaf chlorophyll content; TATL, total area of the top three leaves (cm^2^); ACTL, average chlorophyll content of top three leaves; KGW, 1000-grain weight (g); and KN, kernel number per spike.

### Data analyses of phenotypic traits

Statistical analyses on mean values of the phenotypic traits were performed. Shapiro–Wilk test was performed to test the normal distribution of each trait. Descriptive statistics were estimated and variance analysis was performed using software IBM SPSS 20.0. Origin Pro 2016 was used to draw figures of frequency distribution for the examined traits.

### Association analysis

In total, 14 leaf traits and 2 grain yield trait described above were subjected to association analyses with the SNP markers. The analyses were performed based on the mixed linear model (MLM) with software TASSEL 3.0.124 (http://www.Misogynistic.net/tassel). The probability threshold for a significant trait-marker association was set as 0.001, equivalent to LOD = 3.0. Both Q-Matrix of the population structure and K matrixes used as covariate in MLM analysis were established as described previously [35,39].

## Results

### Statistical analysis of phenotypic traits

Coefficients of variation (CV) among genotypes for all the phenotypic traits were calculated. Mean and CV of the 15 examined traits in three consecutive years were shown in Table 1. All the observed traits showed high CV in the three years. Leaf area was the most genetically variable with high level of CV, ≥ 21.34%, while chlorophyll content showed relatively low level of CV, ≤ 13.22% (Table 1).

**Table 1:** Mean and coefficient of variation for the 15 examined traits of durum wheat in three consecutive years.

Trait ^a^	2015			2016			2017		
	Average	CV (%)	Range	Average	CV (%)	Range	Average	CV (%)	Range
FLL	34.0	15.3	19.8–50.3	30.7	14.7	20.3–46.0	34.2	13.9	21.1–46.2
SLL	37.5	14.1	21.4–52.2	33.6	16.6	20.1–47.6	37.5	12.4	25.7–51.0
TLL	33.7	17.0	19.0–45.1	35.6	14.1	22.3–50.9	35.4	16.0	22.1–48.4
FLW	2.2	15.2	1.4–3.2	2.0	15.3	1.3–2.7	2.2	14.3	1.4–3.1
SLW	2.0	15.7	1.2–2.8	1.9	14.8	1.2–2.6	2.1	14.7	1.3–2.9
TLW	1.7	13.4	1.2–2.3	1.6	14.4	1.1–2.2	1.9	14.4	1.2–2.5
FLA	56.0	25.4	23.8–97.4	46.4	25.8	22.5–82.5	56.1	22.5	28.6–89.8
SLA	56.9	24.6	22.5–96.2	50.4	24.5	23.1–89.4	59.2	22.0	31.7–85.9
TLA	42.5	25.9	16.9–77.6	41.5	27.0	19.6–74.1	49.0	25.9	23.4–75.7
FLCC	45.7	10.8	26.5–58.8	48.8	8.5	36.9–67.2	48.8	8.4	36.9–62.9
SLCC	NA	NA	NA	49.1	10.3	37.9–69.5	49.5	10.4	35.5–65.1
TLCC	NA	NA	NA	45.2	13.1	29.3–57.5	45.6	13.2	26.0–62.8
TATL	156.1	23.2	63.2–261.7	138.3	24.4	68.7–245.9	164.7	21.3	87.4–237.4
ACTL	NA	NA	NA	47.9	9.1	36.0–59.7	48.0	9.2	35.2–60.6
KGW	30.2	25.5	12.8–48.61	35.0	18.6	20.5–57.5	35.4	19.1	13.3–50.4

^a^ FLL, flag leaf length (cm); SLL, second leaf length (cm); TLL, third leaf length (cm); FLW, flag leaf width (cm); SLW, second leaf width (cm); TLW, third leaf width(cm); FLA, flag leaf area (cm^2^); SLA, second leaf area (cm^2^); TLA, third leaf area (cm^2^); FLCC, flag leaf chlorophyll content; SLCC, second leaf chlorophyll content; TLCC, third leaf chlorophyll content; TATL, total area of the top three leaves; ACTL, average chlorophyll content of the top three leaves; KGW, 1000-grain weight (g).

**Fig 1:**
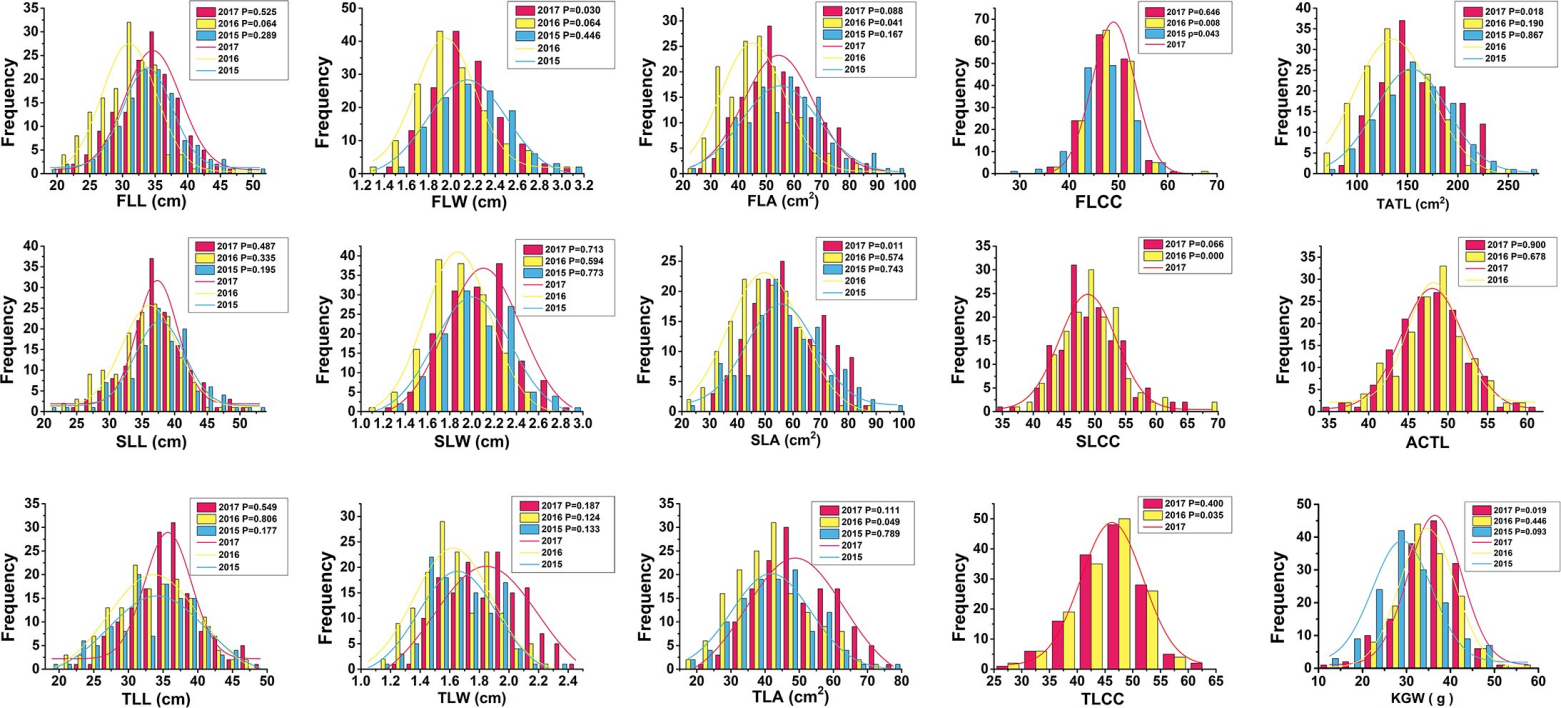
Frequency distribution of the 15 examined canopy leaf traits and 1000-grain weight of durum wheat in three consecutive years. P value of Shapiro–Wilk test for each year was shown, the hypothesis of normal distribution could be accepted when P > 0.05 (significant at P = 0.05), and the trend lines of the accepted normal distribution were shown. FLL, flag leaf length (cm); SLL, second leaf length (cm); TLL, third leaf length (cm); FLW, flag leaf width (cm); SLW, second leaf width (cm); TLW, third leaf width(cm); FLA, flag leaf area (cm^2^); SLA, second leaf area (cm^2^); TLA, third leaf area (cm^2^); FLCC, flag leaf chlorophyll content; SLCC, second leaf chlorophyll content; TLCC, third leaf chlorophyll content; TATL, total area of the top three leaves; ACTL, average chlorophyll content of the top three leaves; KGW, 1000-grain weight (g).

Distribution histograms of the 15 traits were presented in Fig 1. Distributions of the traits were similar in the three years. The Shapiro-Wilk test showed that majority of the observed traits fitted the normal distribution (P ≥ 0.05) (Fig 1). Correlation analysis was performed among the 16 phenotypic traits (Table 2). The results showed that there were 69 highly significant (p < 0.01) and 14 significant (p < 0.05) correlations among the 120 possible correlations. There were significant and positive correlations among the traits of the three canopy leaves. Both the KGW and KN were significantly and positively correlated with chlorophyll content of the canopy leaves, and also with the width and area of canopy leaves (Table 3).

**Table 2:** Correlation coefficients among the 16 observed traits of canopy leaves and grains in durum wheat.

Trait ^a^	FLL	SLL	TLL	FLW	SLW	TLW	FLA	SLA	TLA	FLCC	SLCC	TLCC	TATL	ACTA	KGW
**SLL**	0.901**														
**TLL**	0.709**	0.893**													
**FLW**	0.320**	0.231**	0.105												
**SLW**	0.332**	0.346**	0.312**	0.881**											
**TLW**	0.363**	0.407**	0.410**	0.778**	0.938**										
**FLA**	0.787**	0.675**	0.478**	0.831**	0.761**	0.713**									
**SLA**	0.729**	0.796**	0.711**	0.696**	0.839**	0.835**	0.878**								
**TLA**	0.646**	0.787**	0.861**	0.474**	0.692**	0,798**	0.684**	0.900**							
**FLCC**	-0.084	-0.196*	0.270**	0.189*	0.102	0.069	0.075	-0.034	-0.121						
**SLCC**	-0.076	-0.179*	0.226**	0.212**	0.161*	0.165*	0.100	0.007	-0.037	0.573**					
**TLCC**	-0.128	0.231**	0.263**	0.180*	0.137	0.138	0.047	-0.038	-0.076	0.487**	0.825**				
**TATL**	0.771**	0.802**	0.712**	0.717**	0.818**	0.833**	0.916**	0.989**	0.903**	-0.027	0.018	-0.025			
**ACTL**	-0.097	-0.220**	-0.294**	0.207*	0.147	0.146	0.085	-0.025	-0.089	0.700**	0.940**	0.915**	-0.011		
**KGW**	0.059	0.059	0.057	0.191*	0.227**	0.196*	0.165*	0.184*	0.143	0.243**	0.216**	0.201**	0.167*	0.249**	
**KN**	0.011	-0.011	-0.037	0.249**	0.279**	0.220**	0.167**	0.167**	0.063	0.202*	0.197*	0.259**	0.147	0.250**	0.044

^a^KN, kernel number per spike

*, ** significant at the probability level of 0.05 and 0.01, respectively.

**Table 3:** Number of SNP marker-trait associations in different years for the examined traits.

Year	Trait															
	FLL	SLL	TLL	FLW	SLW	TLW	FLA	SLA	TLA	FLCC	SLCC	TLCC	TATL	ACTL	KGW	Total
2015	9	9	9	9	9	4	9	9	4				4	0	4	13
2016	16	14	14	15	15	15	13	12	13	38	5	2	8	2	5	60
2017	4	4	4	4	4	4	5	4	5	20	6	17	2	17	1	42
Total	28	27	27	28	28	23	27	25	22	38	7	18	14	18	10	92

### Association analysis

Ninety-two SNP markers were found to be significantly associated with the canopy leaf traits and 1000-grain weight in three consecutive years. In the three years from 2015 to 2017, we detected 13, 60 and 42 SNP marker-trait associations, respectively. Among these 115 associations, 23 were repeatedly detected for two years (Table 3).

### Leaf length

Twenty-eight SNP markers were associated with leaf length in the three years. Of these markers, 27 were simultaneously associated with the leaf length of the three canopy leaves. The percentage of the variation explained (PVE) by marker ranged from 7.48% (*BE404339_7_B_649* associated with FLL) to 46.39% (*BE637485_5_B_Y_219* associated with SLL). Three SNP markers, *BE585760_2_A_Y_481* (associated with FLL, SLL and TLL, PVE ≥ 34.0%), *CD452967_5_B_Y_229* (associated with FLL, SLL and TLL, PVE ≥ 36.77%), and *BE637485_5_B_Y_219* (associated with FLL, SLL and TLL, PVE ≥ 46.11%), could explain over 30% of variation (S1 Table).

### Leaf width

Thirty SNP markers were found to be associated with leaf width in the three years. Of these SNP markers, 21 were simultaneously associated with leaf width of the three canopy leaves. For SLW, the lowest PVE was 7.37% for *BE404339_7_B_649* and the highest PVE was 46.33% for *BE637485_5_B_Y_219*. The PVE was over 30% for three SNP markers, *BE585760_2_A_Y_481* (associated with FLW, PVE ≥ 34.64%), *CD452967_5_B_Y_229* (associated with FLW, PVE ≥ 36.76%) and *BE637485_5_B_Y_219* (associated with TLW, PVE ≥ 46.32%) (S1 Table).

### Leaf area

In the three years, 29 marker-trait associations were detected for leaf area. Of these associated markers, 14 were simultaneously associated with four leaf area traits, FLA, SLA, TLA and TATL. The PVE was in a range between 7.67% (*BF474023_3_A_Y_425* associated with FLA) and 45.97% (*BE637485_5_B_Y_219* associated with SLA). There were three SNP markers with PVE > 30%, namely *BE585760_2_A_Y_481* (associated with FLA, PVE ≥ 32.63%), *CD452967_5_B_Y_229* (associated with FLA, PVE ≥ 36.60%), and *BE637485_5_B_Y_219* (associated with FLA, PVE ≥45.52%) (S1 Table).

### Chlorophyll content

In the three consecutive years, we detected 59 marker-trait association pairs for chlorophyll content. Among these associations, 20 were repeatedly detected in two years, and 3 were for two of the two canopy leaves. The 4 associated SNP markers were *BE490384_2_A_Y_544* (associated with FLCC and SLCC in 2016 and 2017), *BE585760_2_A_Y_481* (associated with FLCC and SLCC in 2016 and 2017), *CD452967_5_B_Y_229* (associated with FLCC and SLCC in 2016 and 2017), and *BG274019_2_B_N_260* (associated with FLCC and SLCC in 2016 and 2017) The PVE was in a range between 8.81% (*BF482960_4_B_Y_75* associated with SLCC) and 38.01% (*CD452967_5_B_Y_229* associated with FLCC). Notably, two markers *BE585760_2_A_Y_481* (associated with FLCC, PVE ≥ 34.89%) and *CD452967_5_B_Y_229* (associated with SLCC, PVE ≥ 37.69%) explained more than 30% of the phenotypic variation (S2 Table).

### Grain yield

KGW, the 1000-grain weight, is the one of the key yield components in wheat. A total of 10 SNP marker-trait associations for KGW were detected in the three years from 2015 to 2017. These associations were mainly located on chromosome 1A, 1B, 3A, 4A, 5A, 5B and 7B with PVE of 12.30% - 19.77% (S3 Table). Kernel number per spike (KN) is another key yield component in wheat. Association analysis of the mean KN over four years with the 1366 SNP markers revealed 29 relevant SNP markers. These associations distributed on all except for the 3B chromosomes, and mainly clustered on chromosome 1A, 2A, and 6A. The mean PVE value is 14.28%. The SNP marker *BF482566_6_A_Y_285* had the highest PVE of 32.37% (S4 Table).

## Discussion

The growth and grain yield of cereal crops are closely related to the three canopy leaves [41]. Leaf area and leaf net photosynthesis rate are important traits that affect crop growth and final yield [8]. Chlorophyll content is an important trait reflecting leaf photosynthesis capacity [42]. In wheat, the photosynthesis product after flowering is the main source determining weight gain of the plant and grain yield [43]. At the later stage of wheat growth, the photosynthesis products of the top three leaves contributed up to 80% of grain weight [7]. Therefore, it is very important and necessary to study leaf traits, length, width, area and chlorophyll content, of the three canopy leaves. This study aimed to advance our understanding of the genetic mechanisms underlying morphological traits of the canopy leaves in wheat (*Triticum aestivum* L.). We detected 120 SNP markers associated with the canopy leaf and grain yield traits (S5 Table). These associations would be helpful for us to further understand genetic variation and functional features of the three canopy leaves in wheat.

### SNP markers associated with morphological traits for the three canopy leaves

**Fig 2:**
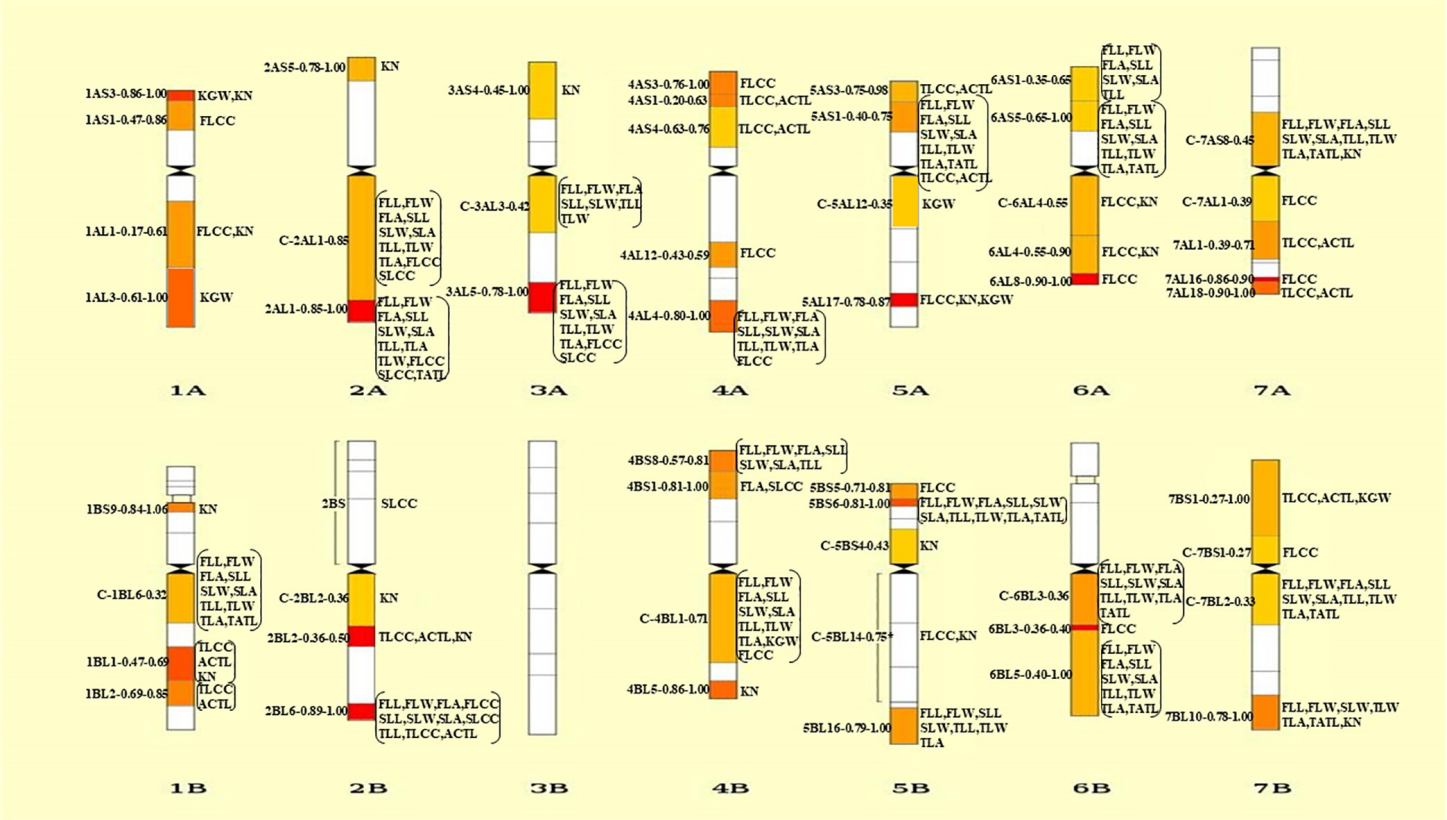
Chromosome bin map of plausible QTL anchored by SNP markers in durum wheat. The relative interval length is indicated on the left of each chromosome and QTL are shown on the right.

A total of 31 SNP markers associated with morphological traits of canopy leaves (leaf length, width and area) were detected on all the 14 chromosomes except for 3B, and 18 of these SNP were associated with all the traits examined. However, the genomic distribution of the associations is uneven, mostly on 2A, 3A, 5B, and 6B chromosomes (Fig 2). The number of SNP markers on chromosome 2 and 6 was larger than that on the other 5 chromosome groups (S1 Table). Gene loci or QTL were previously reported also in the same genome regions of wheat for morphological traits of flag leaf, growth and leaf traits, drought adaptation-related morphological traits, resistance against powdery mildew, and yield-related traits [15–20].

In this study, *BG274019_2_B_N_260* was associated with nine leaf traits, FLL, FLW, FLA, FLCC, SLL, SLW, SLA, SLCC, TLL, and was located in the region of 2BL6-0.89–1.00, a physical interval of about 538.9 Mb (S5 Table). Wu et al. [18] reported that QTL *QFll*.*cau-2B* and *QFla*.*cau-2B* were located in-between markers *wsnp_Ex_c27952_37112702* and *wsnp_Ta_c36_A_1* on chromosome arm 2BL where *BG274019_2_B_N_260* was located. Two associated SNP markers, *BF485396_4_B_N_284* and *BF485396_4_B_N_466*, were detected in the C-4BL 1–0.71 region on chromosome arm 4BL, a physical interval of 438.3 Mb (S5 Table). Of these two markers, *BF485396_4_B_N_284* associated with FLL, FLW, FLA, SLL, SLW, SLW, SLA, TLL, TLW, and TLA. The KGW were located in the same genomic region as QTL (*QFLL-4B*.*2*) reported by Liu et al [20].

The EST-derived SNP marker *BE443540_7_B_N_1397* was associated with all of the 10 morphological traits, and could explain over 11% of the phenotypic variation in the three consecutive years. Physical bin mapping analysis showed that the *BE443540* located in the wheat chromosome bin C-7BL2-0.33 (http://wheat.pw.usda.gov/GG2/index.shtml), and had high homology (E = 1e^-166^) with the putative lipase ROG1 in *Sorghum bicolor* (http://www.ncbi.nlm.nih.gov/). *ROG* plays a key role in regulating plant growth and development, stress resistance and morphogenesis of tissues and organs [44–46]. *BE443540* was also found to be associated with the seedling traits of durum wheat, growth rate of fresh weight and number of leaves in our previous study [34]. Therefore, the gene tagged by SNP marker *BE443540_7_B_N_1397* is evidently involved in the regulation of wheat growth and development.

The EST *BE590521* was developed from wheat 20–45 DAP spike cDNA library, mapped into the wheat chromosome bin C-6BL3-0.36 (http://wheat.pw.usda.gov/GG2/index.shtml), and had very high homology (E = 6e^-108^) with adenine phosphoribosyl transferase (http://www.ncbi.nlm.nih.gov/). The derived SNP marker *BE590521_6_B_N_331* was found to be significantly associated with leaf length and leaf area in the present study (S5 Table). Previous studies have shown that adenine phosphoribosyl transferase is generally the most active in the plant leaves and is involved in the metabolism of cytokinins [47,48]. This shows that our experimental results are very reliable and provide sufficient evidence that this SNP marker can be used in marker-assisted selection for the canopy leaf morphological traits of durum wheat.

The EST for SNP marker *BE606541_6_B_Y_676* was derived from Wheat 5–15 DAP spike cDNA library, it has very high homology (E = 4e^-170^) with DNA damage-inducible protein (http://www.ncbi.nlm.nih.gov/). The SNP marker *BE606541_6_B_Y_676* was shown to be associated with the morphological traits of all the three canopy leaves with R^2^ > 13% in the present study (S1 Table), and also correlated with grain wheat/plant, growth rate of fresh seedling weight, and growth rate for number of leaves in the seedling stage in our previous studies [34,35].

Therefore, some of the genes tagged by the EST-derived SNP markers could confer not only traits of the canopy leaves in matured wheat plants as shown in the present study (S5 Table), but also traits of the seedling and final yield demonstrated in our previous studies on durum wheat [35]. These genes are important for growth, development and formation of the final grain yield in wheat crop. The SNP markers developed in our studies could be helpful not only for marker-assisted breeding for high yield and ideal plant-type, but also for unraveling the genetic mechanism underlying the trait growth, development and final yield construction in wheat.

### Candidate genes for chlorophyll content in the three canopy leaves of durum wheat

Chloroplasts are the most important organelles in plant cell, and are the place not only for photosynthesis but also for biosynthesis of many pre-products. Chloroplasts use chlorophyll to convert light energy into chemical energy through converting CO_2_ and water into sugars[49]. In this study, some SNP markers were found to be specifically associated with enzymes or proteins in chloroplasts.

The EST of *BF293371_7_A_N_1081* was shown very high homology (E = 0.0) with the pre-mRNA-splicing factor ATP-dependent RNA helicase (S5 Table). In plant, ATP-dependent RNA helicases have been found to play an important role in flower meristem decisions, chloroplast differentiation, plant morphogenesis, plant development, etc. [50–52]. In the present study, the RNA helicase could control chlorophyll content in the leaves of durum wheat due to the feasibility controlling chloroplast differentiation. The chlorophyll-associated EST of SNP marker *BE445587_7_A_N_347* showed very high homology (E = 0.0) with the ABC transporter C family member. ABCC (MRP) transporter was initially identified as an ion pump for transporting GS conjugates on vacuoles, which also participate in other physiological processes, such as detoxification in cells, transport of chlorophyll metabolites, and regulation of ion channels [53]. Meanwhile, we found another PDR-type ABC transporter (*PDR1*) with a SNP marker *BM137384_5_A_444* in this study. Previous study demonstrated that *TaPDR1* was associated with gibberellic disease[54]. Therefore, the transportation of chlorophyll in wheat might be related with some particular class of ABC transporters.

### The pleiotropy of candidate genes conferring the canopy leaf traits and yield

In nature, pleiotropy is the phenomenon that a single gene may simultaneously affect several phenotypic traits. In the process of organism development, many biochemical reactions are interdependent. The target gene controlling the specific trait may influence a series of biochemical reactions. In the present study, many genes tagged by SNP markers controlled multiple canopy leaf traits (S5 Table), implying the common phenomenon of pleiotropy. Coincidentally, the pleiotropy also occurs in the various development stages in durum wheat (S6 Table). Several important pleiotropic loci were further identified by analysis of EST sequences. For example, the EST of *BE405834_1_B_Y_216* showed very high homology (E = 0.0) (http://www.ncbi.nlm.nih.gov/) with the soluble inorganic pyrophosphatase (S5 Table). The enzyme is widely distributed in nature and participates in the hydrolysis of pyrophosphate formed in various metabolic pathways, releases energy, provides energy for various physiological mechanisms, and regulates the growth and development of organisms [55–57]. The enzyme in durum wheat not only participates in the regulation of the number of leaves and fresh weight at the seedling stage, but also affects the morphological traits of the three canopy leaves and the grain weight per plant. These evidences fully demonstrate that the enzyme's role in durum wheat is multi- functional.

The EST of SNP marker *BE443538_5_A_1436* had very high homology (E = 0.0) with the LIM domain-containing protein. LIM protein family mediates protein-protein interactions and has one or more zinc finger structures in its molecular structure [58]. The family members are widely involved in the development of a variety of cells and the regulation of differentiation and transcription [59,60]. In our previous studies, this LIM protein contributed to the number of leaves and fresh weight of durum wheat at the seedling stage [34]. At the adult stage, this LIM protein was associated with the morphological traits of the three canopy leaves and rachis internode length of main spike [35]. Moreover, it also affects grain number per plant and grain weight per plant [35]. Thus the LIM protein as a multi-functional gene plays an important regulatory role in cell differentiation, organ development, and cytoskeletal formation at different growth stages of durum wheat.

About 80% of wheat yield is accumulated through photosynthesis in canopy leaves [7]. Chlorophyll content and leaf size are key factors for photosynthesis in wheat [10]. We detected a significant association between the two SNP markers, *BE490384_2_A_Y_544* and *BE585760_2_A_Y_481*, in the 2AL1-0.85–1.00 region of chromosome 2A and the size and chlorophyll content of the canopy leaf (S5 Table). One SNP marker, *BE517711_5_B_49* on chromosome 5B, was significantly associated with FLCC. The physical genomic location for this association was 5B: 487221967–487222137. A major QTL for FLL (*QFll*.*sicau-5B*) [19] was located on chromosome 5B and was 4.9 Mb from the SNP marker, *BE517711_5_B_49*. Given that SNP markers for canopy leaf-related traits co-localized in the same region, the region should contain a major QTL with pleiotropic effects or multiple linked SNP markers.

## Conclusions

We demonstrated significant positive correlations among morphological traits (leaf length, width and area), and negative correlations between the morphological traits and the chlorophyll content of the canopy leaves in durum wheat (Table 2). There was a significant positive correlation between the yield traits and leaf width and chlorophyll content of canopy leaves (Table 2). Through association analyses on 16 canopy leaf and yield traits with 1366 EST-derived SNP markers, 120 SNP marker-trait associations were identified (S5 Table). Some of the SNP markers were associated with multiple traits due to the pleiotropic effects (S5 and S6 Table). The results might be helpful for understanding the genetic mechanism controlling leaf morphology and photosynthesis, and marker-assisted breeding for ideal plant-type and high photosynthesis efficiency in durum wheat.

## Supporting information

S1 Table Significant associations between morphological trait of the three canopy leaves and SNP marker in durum wheat. LL, leaf length (cm); LW, leaf width (cm); LA, leaf area (cm^2^).
(DOCX)

S2 Table Significant associations between chlorophyll contentin the three canopy leaves and SNP maekers in durum wheat. FLCC, flag leaf chlorophyll content; SLCC, second leaf chlorophyll content; TLCC, third leaf chlorophyll content; ACTL: average chlorophyll content of top three leaves.
(DOCX)

S3 Table Significant associations between 1000-grain weight and SNP markers in durum wheat. KGW, 1000-grain weight (g).
(DOCX)

S4 Table Significant association between kernel number per spike and SNP markers in durum wheat. KN, kernel number per spike.
(DOCX)

S5 Table The plausible functions in the homologous sequences of associated EST. a: Overlapping gene by blast from http://www.ensembl.org/; b: Gene function and the homologous EST correspond to the best hit detected by blast from http://www.ncbi.nlm.nih.gov/; c:FLL, flag leaf length (cm); SLL, second leaf length (cm); TLL, third leaf length (cm);FLW, flag leaf width (cm); SLW, second leaf width (cm); TLW, third leaf width(cm); FLA, flag leaf area (cm2); SLA, second leaf area (cm2); TLA, third leaf area (cm2); FLCC, flag leaf chlorophyll content; SLCC, second leaf chlorophyll content; TLCC, third leaf chlorophyll content;TATL, total area of the top three leaves; ACTL, average chlorophyll content of the top three leaves; KGW, 1000-grain weight (g); KN, kernel number per spike.
(XLSX)

S6 Table SNP markers associated with multiple traits in durum wheat. GNP, grain number per plant; GWP, grain weight per plant (g); RLMS, rachis internode length of main spike (cm); KGW, 1000-grain weight (g); SMS, number of spikelets on main spike; FW, fresh weight (g); NL, number of leaves; GRFW, growth rate of fresh weight; GRNL, growth rate for number of leaves; LA, leaf area (cm2); GRNR, growth rate for number of roots; GRLA, growth rate of leaf area.
(DOCX)
