# Supplementary material for: Associations of canopy leaf traits with SNP markers in durum wheat (Triticum turgidum L. durum (Desf.))
Source: PLoS One. 2018 Oct 23;13(10):e0206226. doi: 10.1371/journal.pone.0206226 (PMC6198983; doi:10.1371/journal.pone.0206226)
Supplement: S1 Table — LL, leaf length (cm); LW, leaf width (cm); LA, leaf area (cm2). (DOCX) [file pone.0206226.s001.docx]

**S1 Table. Significant associations between morphological trait of the three canopy leaves and SNP markers in durum wheat.**

| Trait ^a^ | SNP markers | Chromosome bin | Flag leaf | | Second leaf | | Third leaf | |
| --- | --- | --- | --- | --- | --- | --- | --- | --- |
|  |  |  | p | R^2^ | p | R^2^ | p | R^2^ |
| LA | BE445121_1_A_Y_552 | 1A | 0.0000 | 0.1397 | 0.0000 | 0.1356 | 0.0000 | 0.1395 |
| LL | BE445121_1_A_Y_552 | 1A | 0.0000 | 0.1538 | 0.0000 | 0.1518 | 0.0000 | 0.1529 |
| LW | BE445121_1_A_Y_552 | 1A | 0.0000 | 0.1583 | 0.0000 | 0.1583 | 0.0000 | 0.1583 |
| LA | BE405834_1_B_Y_216 | 1B | 0.0000 | 0.1234 | 0.0000 | 0.1245 |  |  |
| LL | BE405834_1_B_Y_216 | 1B | 0.0000 | 0.127 | 0.0000 | 0.1273 | 0.0000 | 0.1281 |
| LW | BE405834_1_B_Y_216 | 1B | 0.0000 | 0.1271 | 0.0000 | 0.1271 |  |  |
| LA | BE490384_2_A_Y_544 | 2AL1-0.85-1.00 | 0.0000 | 0.1801 | 0.0000 | 0.166 | 0.0009 | 0.0984 |
| LL | BE490384_2_A_Y_544 | 2AL1-0.85-1.00 | 0.0000 | 0.1608 | 0.0000 | 0.165 | 0.0000 | 0.1645 |
| LW | BE490384_2_A_Y_544 | 2AL1-0.85-1.00 | 0.0000 | 0.1691 | 0.0000 | 0.1548 |  |  |
| LA | BE499251_2_A_N_239 | 2AL1-0.85-1.00 | 0.0000 | 0.1397 | 0.0000 | 0.1356 | 0.0000 | 0.1395 |
| LL | BE499251_2_A_N_239 | 2AL1-0.85-1.00 | 0.0000 | 0.1538 | 0.0000 | 0.1518 | 0.0000 | 0.1529 |
| LW | BE499251_2_A_N_239 | 2AL1-0.85-1.00 | 0.0000 | 0.1583 | 0.0000 | 0.1583 | 0.0000 | 0.1583 |
| LA | BG274019_2_B_N_260 | 2BL6-0.89-1.00 | 0.0000 | 0.1728 | 0.0000 | 0.1608 |  |  |
| LL | BG274019_2_B_N_260 | 2BL6-0.89-1.00 | 0.0000 | 0.1912 | 0.0000 | 0.1696 | 0.0000 | 0.1673 |
| LW | BG274019_2_B_N_260 | 2BL6-0.89-1.00 | 0.0000 | 0.1609 | 0.0000 | 0.1609 |  |  |
| LA | BE517914_3_A_Y_81 | 3A | 0.0000 | 0.1398 | 0.0001 | 0.1356 | 0.0000 | 0.1395 |
| LL | BE517914_3_A_Y_81 | 3A | 0.0000 | 0.1538 | 0.0000 | 0.1518 | 0.0000 | 0.1529 |
| LW | BE517914_3_A_Y_81 | 3A | 0.0000 | 0.1583 | 0.0000 | 0.1583 | 0.0000 | 0.1583 |
| LA | BF428994_3_A_N_324 | 3AL5-0.78-1.00 | 0.0007 | 0.0993 | 0.0008 | 0.0977 | 0.0006 | 0.1005 |
| LL | BF428994_3_A_N_324 | 3AL5-0.78-1.00 | 0.0005 | 0.104 | 0.0005 | 0.1034 | 0.0005 | 0.1056 |
| LW | BF428994_3_A_N_324 | 3AL5-0.78-1.00 | 0.0005 | 0.1049 | 0.0005 | 0.1049 | 0.0005 | 0.1049 |
| LA | BF474410_4_A_394 | 4AL4-0.80-1.00 | 0.0002 | 0.1398 | 0.0002 | 0.1357 | 0.0002 | 0.1397 |
| LL | BF474410_4_A_394 | 4AL4-0.80-1.00 | 0.0001 | 0.1538 | 0.0001 | 0.1519 | 0.0001 | 0.153 |
| LW | BF474410_4_A_394 | 4AL4-0.80-1.00 | 0.0001 | 0.1583 | 0.0001 | 0.1583 | 0.0001 | 0.1583 |
| LA | BF485396_4_B_N_284 | C-4BL1-0.71 | 0.0002 | 0.1398 | 0.0002 | 0.1356 | 0.0002 | 0.1396 |
| LL | BF485396_4_B_N_284 | C-4BL1-0.71 | 0.0001 | 0.1538 | 0.0001 | 0.1518 | 0.0001 | 0.1529 |
| LW | BF485396_4_B_N_284 | C-4BL1-0.71 | 0.0001 | 0.1583 | 0.0001 | 0.1583 | 0.0001 | 0.1583 |
| LA | BF482960_4_B_Y_75 | 4BS1-0.81-1.00 | 0.0007 | 0.0809 |  |  |  |  |
| LA | BE442666_4_B_Y_327 | 4BS8-0.57-0.81 | 0.0001 | 0.1233 | 0.0000 | 0.1246 |  |  |
| LL | BE442666_4_B_Y_327 | 4BS8-0.57-0.81 | 0.0000 | 0.1259 | 0.0000 | 0.1264 | 0.0000 | 0.1272 |
| LW | BE442666_4_B_Y_327 | 4BS8-0.57-0.81 | 0.0000 | 0.1258 | 0.0000 | 0.1258 |  |  |
| LA | BE443538_5_A_1436 | 5AS1-0.40-0.75 | 0.0000 | 0.1448 | 0.0000 | 0.1458 | 0.0004 | 0.0841 |
| LL | BE443538_5_A_1436 | 5AS1-0.40-0.75 | 0.0000 | 0.1482 | 0.0000 | 0.1485 | 0.0000 | 0.1495 |
| LW | BE443538_5_A_1436 | 5AS1-0.40-0.75 | 0.0000 | 0.1485 | 0.0000 | 0.1486 | 0.0005 | 0.0833 |
| LA | CD452967_5_B_Y_229 | 5B | 0.0000 | 0.3660 | 0.0000 | 0.3788 | 0.0000 | 0.1957 |
| LL | CD452967_5_B_Y_229 | 5B | 0.0000 | 0.3677 | 0.0000 | 0.3720 | 0.0000 | 0.3738 |
| LW | CD452967_5_B_Y_229 | 5B | 0.0000 | 0.3676 | 0.0000 | 0.3684 | 0.0000 | 0.1595 |
| LL | BE403710_5_B_468 | 5BL16-0.79-1.00 | 0.0005 | 0.1216 | 0.0006 | 0.1205 | 0.0005 | 0.1221 |
| LW | BE403710_5_B_468 | 5BL16-0.79-1.00 | 0.0004 | 0.1268 | 0.0004 | 0.1269 | 0.0004 | 0.1270 |
| LA | BE403710_5_B_468 | 5BL16-0.79-1.00 |  |  |  |  | 0.0009 | 0.1125 |
| LA | BE637485_5_B_Y_219 | 5BS6-0.81-1.00 | 0.0000 | 0.4552 | 0.0000 | 0.4597 | 0.0000 | 0.4588 |
| LL | BE637485_5_B_Y_219 | 5BS6-0.81-1.00 | 0.0000 | 0.4611 | 0.0000 | 0.4639 | 0.0000 | 0.4631 |
| LW | BE637485_5_B_Y_219 | 5BS6-0.81-1.00 | 0.0000 | 0.4633 | 0.0000 | 0.4633 | 0.0000 | 0.4632 |
| LA | BE606541_6_B_Y_676 | 6B | 0.0000 | 0.1323 | 0.0000 | 0.1330 |  |  |
| LL | BE606541_6_B_Y_676 | 6B | 0.0000 | 0.1348 | 0.0000 | 0.1349 | 0.0000 | 0.1355 |
| LW | BE606541_6_B_Y_676 | 6B | 0.0000 | 0.1345 | 0.0000 | 0.1346 |  |  |
| LA | BE591957_6_A_N_163 | 6AS5-0.65-1.00 | 0.0000 | 0.1397 | 0.0000 | 0.1356 | 0.0000 | 0.1395 |
| LL | BE591957_6_A_N_163 | 6AS5-0.65-1.00 | 0.0000 | 0.1538 | 0.0000 | 0.1518 | 0.0000 | 0.1529 |
| LW | BE591957_6_A_N_163 | 6AS5-0.65-1.00 | 0.0000 | 0.1583 | 0.0000 | 0.1583 | 0.0000 | 0.1583 |
| LA | BE499711_6_B_Y_294 | 6B | 0.0000 | 0.1397 | 0.0000 | 0.1356 | 0.0000 | 0.1395 |
| LL | BE499711_6_B_Y_294 | 6B | 0.0000 | 0.1538 | 0.0000 | 0.1518 | 0.0000 | 0.1529 |
| LW | BE499711_6_B_Y_294 | 6B | 0.0000 | 0.1583 | 0.0000 | 0.1583 | 0.0000 | 0.1583 |
| LA | BE591777_6_B_244 | 6BL5-0.40-1.00 | 0.0000 | 0.1398 | 0.0001 | 0.1356 | 0.0000 | 0.1395 |
| LL | BE591777_6_B_244 | 6BL5-0.40-1.00 | 0.0000 | 0.1538 | 0.0000 | 0.1518 | 0.0000 | 0.1529 |
| LW | BE591777_6_B_244 | 6BL5-0.40-1.00 | 0.0000 | 0.1583 | 0.0000 | 0.1583 | 0.0000 | 0.1583 |
| LA | BE445587_7_A_N_347 | 7AS8-0.45-0.89* | 0.0006 | 0.0911 | 0.0006 | 0.0919 |  |  |
| LL | BE445587_7_A_N_347 | 7AS8-0.45-0.89* | 0.0005 | 0.0940 | 0.0005 | 0.0942 | 0.0005 | 0.0946 |
| LW | BE445587_7_A_N_347 | 7AS8-0.45-0.89* | 0.0005 | 0.0944 | 0.0005 | 0.0945 |  |  |
| LL | BE404339_7_B_649 | 7BL10-0.78-1.00 | 0.0009 | 0.0748 |  |  |  |  |
| LW | BE404339_7_B_649 | 7BL10-0.78-1.00 | 0.0001 | 0.0738 | 0.0001 | 0.0737 | 0.0001 | 0.0738 |
| LA | BE445506_7_B_Y_355 | 7BL10-0.78-1.00 |  |  |  |  | 0.0000 | 0.3278 |
| LW | BE445506_7_B_Y_355 | 7BL10-0.78-1.00 |  |  |  |  | 0.0000 | 0.2745 |
| LA | BE518436_7_B_Y_671 | 7BS |  |  |  |  | 0.0000 | 0.1564 |
| LW | BE518436_7_B_Y_671 | 7BS |  |  |  |  | 0.0001 | 0.1317 |
| LA | BG314205_1_B_33 | C-1BL6-0.32 | 0.0000 | 0.1397 | 0.0000 | 0.1408 | 0.0001 | 0.107 |
| LL | BG314205_1_B_33 | C-1BL6-0.32 | 0.0000 | 0.1429 | 0.0000 | 0.1432 | 0.0000 | 0.1442 |
| LW | BG314205_1_B_33 | C-1BL6-0.32 | 0.0000 | 0.1432 | 0.0000 | 0.1432 | 0.0001 | 0.1061 |
| LA | BE403516_2_A_Y_254 | C-2AL1-0.85 | 0.0005 | 0.0808 | 0.0007 | 0.0771 | 0.0007 | 0.0772 |
| LL | BE403516_2_A_Y_254 | C-2AL1-0.85 | 0.0004 | 0.0871 | 0.0004 | 0.0854 | 0.0004 | 0.0844 |
| LW | BE403516_2_A_Y_254 | C-2AL1-0.85 | 0.0003 | 0.089 | 0.0003 | 0.089 | 0.0003 | 0.089 |
| LA | BE585760_2_A_Y_481 | C-2AL1-0.85 | 0.0000 | 0.3263 | 0.0000 | 0.3315 | 0.0000 | 0.1854 |
| LL | BE585760_2_A_Y_481 | C-2AL1-0.85 | 0.0000 | 0.3400 | 0.0000 | 0.3408 | 0.0000 | 0.3399 |
| LW | BE585760_2_A_Y_481 | C-2AL1-0.85 | 0.0000 | 0.3464 | 0.0000 | 0.3469 | 0.0001 | 0.1504 |
| LA | BF145580_2_A_107 | C-2AL1-0.85 | 0.0004 | 0.0775 | 0.0004 | 0.078 |  |  |
| LL | BF145580_2_A_107 | C-2AL1-0.85 | 0.0003 | 0.0796 | 0.0003 | 0.0798 | 0.0003 | 0.0805 |
| LW | BF145580_2_A_107 | C-2AL1-0.85 | 0.0003 | 0.0799 | 0.0003 | 0.0799 |  |  |
| LA | BF474023_3_A_Y_425 | C-3AL3-0.42 | 0.0007 | 0.0767 |  |  |  |  |
| LL | BF474023_3_A_Y_425 | C-3AL3-0.42 | 0.0005 | 0.0819 | 0.0006 | 0.0803 | 0.0006 | 0.0796 |
| LW | BF474023_3_A_Y_425 | C-3AL3-0.42 | 0.0005 | 0.0833 | 0.0005 | 0.0832 | 0.0005 | 0.0832 |
| LA | BE590521_6_B_N_331 | C-6BL3-0.36 | 0.0000 | 0.1448 | 0.0000 | 0.1458 | 0.0004 | 0.0841 |
| LL | BE590521_6_B_N_331 | C-6BL3-0.36 | 0.0000 | 0.1482 | 0.0000 | 0.1485 | 0.0000 | 0.1495 |
| LW | BE590521_6_B_N_331 | C-6BL3-0.36 | 0.0000 | 0.1485 | 0.0000 | 0.1486 | 0.0005 | 0.0833 |
| LA | BE471272_7_A_423 | C-7AS8-0.45 | 0.0000 | 0.1397 | 0.0000 | 0.1356 | 0.0000 | 0.1395 |
| LL | BE471272_7_A_423 | C-7AS8-0.45 | 0.0000 | 0.1538 | 0.0000 | 0.1518 | 0.0000 | 0.1529 |
| LW | BE471272_7_A_423 | C-7AS8-0.45 | 0.0000 | 0.1583 | 0.0000 | 0.1583 | 0.0000 | 0.1583 |
| LA | BE443540_7_B_N_1397 | C-7BL2-0.33 | 0.0000 | 0.1782 | 0.0000 | 0.1794 | 0.0007 | 0.1175 |
| LL | BE443540_7_B_N_1397 | C-7BL2-0.33 | 0.0000 | 0.1817 | 0.0000 | 0.1821 | 0.0000 | 0.1831 |
| LW | BE443540_7_B_N_1397 | C-7BL2-0.33 | 0.0000 | 0.1818 | 0.0000 | 0.1819 | 0.0007 | 0.1164 |

^a^ LL, leaf length (cm); LW, leaf width (cm); LA, leaf area (cm^2^).
