# Supplementary material for: Associations of canopy leaf traits with SNP markers in durum wheat (Triticum turgidum L. durum (Desf.))
Source: PLoS One. 2018 Oct 23;13(10):e0206226. doi: 10.1371/journal.pone.0206226 (PMC6198983; doi:10.1371/journal.pone.0206226)
Supplement: S2 Table — FLCC, flag leaf chlorophyll content; SLCC, second leaf chlorophyll content; TLCC, third leaf chlorophyll content; ACTL: average chlorophyll content of top three leaves. (DOCX) [file pone.0206226.s002.docx]

**S2 Table. Significant associations between chlorophyll content in the three canopy leaves and SNP markers in durum wheat.**

| Trait ^a^ | SNP markers | Chromosome bin | 2016 | | 2017 | |
| --- | --- | --- | --- | --- | --- | --- |
|  |  |  | p | R^2^ | p | R^2^ |
| FLCC | BF484606_1_A_390 | 1A | 0.0001 | 0.1067 | 0.0004 | 0.0899 |
| FLCC | BG274119_1_A_Y_221 | 1A | 0.0005 | 0.1067 |  |  |
| FLCC | BF292414_1_A_78 | 1AL1-0.17-0.61 | 0.0005 | 0.1062 |  |  |
| FLCC | BF485305_1_A_Y_29 | 1AL1-0.17-0.61 | 0.0005 | 0.1068 |  |  |
| FLCC | BF474569_1_A_Y_382 | 1AS1-0.47-0.86 | 0.0001 | 0.1067 | 0.0004 | 0.0899 |
| FLCC | BE399980_1_A_Y_557 | 1AS1-0.47-0.87 | 0.0005 | 0.1071 |  |  |
| FLCC | BF484496_1_B_N_150 | 1B | 0.0001 | 0.1067 | 0.0004 | 0.0899 |
| TLCC | BF478690_1_B_Y_78 | 1BL1-0.47-0.69 |  |  | 0.0007 | 0.1203 |
| ACTL | BF478690_1_B_Y_78 | 1BL1-0.47-0.69 |  |  | 0.0007 | 0.1205 |
| TLCC | BG313767_1_B_107 | 1BL1-0.47-0.85* |  |  | 0.0002 | 0.1167 |
| ACTL | BG313767_1_B_107 | 1BL1-0.47-0.85* |  |  | 0.0002 | 0.1177 |
| TLCC | BG263233_1_B_825 | 1BL2-0.69-0.85 | 0.0004 | 0.1121 | 0.0001 | 0.1402 |
| ACTL | BG263233_1_B_825 | 1BL2-0.69-0.85 | 0.0004 | 0.1112 |  |  |
| ACTL | BG263233_1_B_825 | 1BL2-0.69-0.85 |  |  | 0.0000 | 0.1414 |
| FLCC | BE490384_2_A_Y_544 | 2AL1-0.85-1.00 | 0.0000 | 0.1507 | 0.0000 | 0.1497 |
| SLCC | BE490384_2_A_Y_544 | 2AL1-0.85-1.00 | 0.0000 | 0.1524 | 0.0000 | 0.1512 |
| FLCC | BE490763_2_A_1462 | 2AL1-0.85-1.00 | 0.0005 | 0.1068 |  |  |
| TLCC | BF202975_2_B_280 | 2BL2-0.36-0.50 |  |  | 0.0002 | 0.1194 |
| ACTL | BF202975_2_B_280 | 2BL2-0.36-0.50 |  |  | 0.0002 | 0.1199 |
| FLCC | BG274019_2_B_N_260 | 2BL6-0.89-1.00 | 0.0000 | 0.1696 | 0.0000 | 0.1645 |
| FLCC | BG274019_2_B_N_260 | 2BL6-0.89-1.00 | 0.0000 | 0.1639 | 0.0000 | 0.1671 |
| TLCC | BQ172173_2_B_Y_157 | 2BL6-0.89-1.00 |  |  | 0.0007 | 0.1187 |
| ACTL | BQ172173_2_B_Y_157 | 2BL6-0.89-1.00 |  |  | 0.0005 | 0.1265 |
| SLCC | BE444144_2_B_92 | 2BS |  |  | 0.0009 | 0.0997 |
| FLCC | BQ167580_3_A_Y_342 | 3A | 0.0002 | 0.1432 | 0.0007 | 0.122 |
| SLCC | BE404374_3_A_Y_612 | 3AL5-0.78-1.00 | 0.0008 | 0.1009 |  |  |
| FLCC | BF428994_3_A_N_324 | 3AL5-0.78-1.00 | 0.0005 | 0.1081 |  |  |
| FLCC | BG262734_3_A_N_190 | 3AL5-0.78-1.00 | 0.0006 | 0.1057 |  |  |
| TLCC | BE444858_4_A_Y_87 | 4A |  |  | 0.0002 | 0.1195 |
| TLCC | BE591861_4_A_Y_848 | 4A |  |  | 0.0002 | 0.1195 |
| ACTL | BE444858_4_A_Y_87 | 4A |  |  | 0.0002 | 0.12 |
| ACTL | BE591861_4_A_Y_848 | 4A |  |  | 0.0002 | 0.12 |
| FLCC | BE443500_4_A_N_610 | 4AL12-0.43-0.59 | 0.0001 | 0.1062 | 0.0004 | 0.0895 |
| FLCC | BE494023_4_A_N_380 | 4AL4-0.80-1.00 | 0.0001 | 0.1067 | 0.0004 | 0.0899 |
| TLCC | BE443973_4_A_105 | 4AS1-0.20-0.63 |  |  | 0.0002 | 0.1195 |
| ACTL | BE443973_4_A_105 | 4AS1-0.20-0.63 |  |  | 0.0002 | 0.1200 |
| FLCC | BE438226_4_A_N_681 | 4AS3-0.76-1.00 | 0.0001 | 0.1067 | 0.0004 | 0.0899 |
| FLCC | BF483551_4_A_N_203 | 4AS3-0.76-1.00 | 0.0005 | 0.1066 |  |  |
| TLCC | BE425301_4_A_Y_160 | 4AS4-0.63-0.76 |  |  | 0.0001 | 0.1070 |
| FLCC | BF485396_4_B_N_466 | C-4BL-0.71 | 0.0006 | 0.1055 |  |  |
| ACTL | BE425301_4_A_Y_160 | 4AS4-0.63-0.76 |  |  | 0.0001 | 0.1068 |
| SLCC | BF482960_4_B_Y_75 | 4BS1-0.81-1.00 |  |  | 0.0004 | 0.0881 |
| FLCC | BM137384_5_A_444 | 5AL17-0.78-0.87 | 0.0001 | 0.1067 | 0.0004 | 0.0899 |
| TLCC | BG607308_5_A_Y_101 | 5AL17-0.78-0.87 |  |  | 0.0001 | 0.1530 |
| ACTL | BG607308_5_A_Y_101 | 5AL17-0.78-0.87 |  |  | 0.0000 | 0.1668 |
| TLCC | BE444644_5_A_Y_287 | 5AS1-0.40-0.75 |  |  | 0.0002 | 0.1166 |
| ACTL | BE444644_5_A_Y_287 | 5AS1-0.40-0.75 |  |  | 0.0002 | 0.1176 |
| TLCC | BF201102_5_A_Y_154 | 5AS3-0.75-0.98 |  |  | 0.0006 | 0.1209 |
| ACTL | BF201102_5_A_Y_154 | 5AS3-0.75-0.98 |  |  | 0.0006 | 0.1212 |
| FLCC | CD452967_5_B_Y_229 | 5B | 0.0000 | 0.3801 | 0.0000 | 0.3783 |
| SLCC | CD452967_5_B_Y_229 | 5B | 0.0000 | 0.3769 | 0.0000 | 0.3800 |
| FLCC | BE517711_5_B_49 | 5B | 0.0001 | 0.1067 | 0.0004 | 0.0899 |
| FLCC | BI479113_5_B_534 | 5BS5-0.71-0.81 | 0.0001 | 0.1073 | 0.0003 | 0.0905 |
| FLCC | BE426214_6_A_N_191 | 6AL4-0.55-0.90 | 0.0001 | 0.1067 | 0.0004 | 0.0899 |
| FLCC | BF294007_6_A_Y_116 | 6AL8-0.90-1.00 | 0.0001 | 0.1464 | 0.0005 | 0.1259 |
| FLCC | BE442905_6_B_N_1225 | 6B | 0.0001 | 0.1067 | 0.0004 | 0.0899 |
| FLCC | BQ159615_6_B_N_189 | 6B | 0.0005 | 0.1068 |  |  |
| FLCC | BQ169448_6_B_252 | 6B | 0.0006 | 0.1061 |  |  |
| FLCC | BE438495_6_B_Y_71 | 6BL3-0.36-0.40 | 0.0004 | 0.1108 |  |  |
| FLCC | BF292614_6_A_189 | 6AL8-0.90-1.00 | 0.0005 | 0.1068 |  |  |
| FLCC | BF293371_7_A_N_1081 | 7A | 0.0001 | 0.1066 | 0.0004 | 0.0899 |
| TLCC | BE406943_7_A_Y_313 | 7AL1-0.39-0.71 | 0.0007 | 0.1210 |  |  |
| TLCC | BF482529_7_A_304 | 7AL1-0.39-0.71 |  |  | 0.0003 | 0.1318 |
| ACTL | BE406943_7_A_Y_313 | 7AL1-0.39-0.71 | 0.0007 | 0.1204 |  |  |
| ACTL | BF482529_7_A_304 | 7AL1-0.39-0.71 |  |  | 0.0001 | 0.1522 |
| FLCC | BF474379_7_A_83 | 7AL16-0.86-0.90 | 0.0005 | 0.1066 |  |  |
| TLCC | BQ169669_7_A_Y_378 | 7AL18-0.90-1.00 |  |  | 0.0003 | 0.1317 |
| ACTL | BQ169669_7_A_Y_378 | 7AL18-0.90-1.00 |  |  | 0.0003 | 0.1328 |
| TLCC | BE445587_7_A_N_347 | 7AS8-0.45-0.89* |  |  | 0.0002 | 0.1176 |
| ACTL | BE445587_7_A_N_347 | 7AS8-0.45-0.89* |  |  | 0.0002 | 0.1188 |
| TLCC | BF474552_7_B_Y_127 | 7BS1-0.27-1.00 |  |  | 0.0002 | 0.1166 |
| TLCC | CD452629_7_B_58 | 7BS1-0.27-1.00 |  |  | 0.0008 | 0.1181 |
| ACTL | BF474552_7_B_Y_127 | 7BS1-0.27-1.00 |  |  | 0.0002 | 0.1177 |
| ACTL | CD452629_7_B_58 | 7BS1-0.27-1.00 |  |  | 0.0007 | 0.1195 |
| FLCC | BE585760_2_A_Y_481 | C-2AL1-0.85 | 0.0000 | 0.3569 | 0.0000 | 0.3489 |
| SLCC | BE585760_2_A_Y_481 | C-2AL1-0.85 | 0.0000 | 0.3551 | 0.0000 | 0.3510 |
| FLCC | BE591423_5_B_Y_580 | C-5BL14-0.75* | 0.0001 | 0.1067 | 0.0004 | 0.0899 |
| FLCC | CD453593_6_A_N_238 | C-6AL4-0.55 | 0.0005 | 0.1067 |  |  |
| FLCC | BE424174_7_A_Y_193 | C7A | 0.0001 | 0.1055 | 0.0004 | 0.089 |
| FLCC | BE494028_7_A_Y_108 | C-7AL1-0.39 | 0.0004 | 0.1093 |  |  |
| FLCC | BG604857_7_B_N_74 | C-7BS1-0.27 | 0.0005 | 0.1068 |  |  |

^a^ FLCC, flag leaf chlorophyll content; SLCC, second leaf chlorophyll content; TLCC, third leaf chlorophyll content; ACTL: average chlorophyll content of top three leaves.
