# Supplementary material for: Associations of canopy leaf traits with SNP markers in durum wheat (Triticum turgidum L. durum (Desf.))
Source: PLoS One. 2018 Oct 23;13(10):e0206226. doi: 10.1371/journal.pone.0206226 (PMC6198983; doi:10.1371/journal.pone.0206226)
Supplement: S3 Table — KGW, 1000-grain weight (g). (DOCX) [file pone.0206226.s003.docx]

**S3 Table. Significant associations between 1000-grain weight and SNP markers in durum wheat.**

| Trait ^a^ | SNP markers | Chromosome bin | 2015 | | 2016 | | 2017 | |
| --- | --- | --- | --- | --- | --- | --- | --- | --- |
|  |  |  | p | R^2^ | p | R^2^ | p | R^2^ |
| KGW | BE405060_5_A_N_606 | C-5AL12-0.35 | 0.0000 | 0.1977 |  |  |  |  |
| KGW | BF485396_4_A_N_284 | 4AS4-0.63-0.76 | 0.0000 | 0.1572 |  |  |  |  |
| KGW | BG314157_1_A_127 | 1AL3-0.61-1.00 | 0.0000 | 0.1553 |  |  |  |  |
| KGW | BE426222_3_A_68 | 3A | 0.0000 | 0.1539 |  |  |  |  |
| KGW | BE403322_1_A_Y_426 | 1AL3-0.61-1.00 |  |  | 0.0000 | 0.1463 |  |  |
| KGW | BE406609_5_A_65 | 5AL17-0.78-0.87 |  |  | 0.0000 | 0.1372 |  |  |
| KGW | BE586140_1_A_Y_220 | 1AS3-0.86-1.00 |  |  | 0.0000 | 0.1336 |  |  |
| KGW | BE591682_1_B_Y_190 | 1B |  |  | 0.0002 | 0.1411 |  |  |
| KGW | BG604507_4_B_383 | 4B |  |  | 0.0006 | 0.1230 |  |  |
| KGW | BE405835_7_B_Y_166 | 7BS1-0.27-1.00 |  |  |  |  | 0.0000 | 0.1887 |

^a^ KGW, 1000-grain weight (g).
