# Supplementary material for: Associations of canopy leaf traits with SNP markers in durum wheat (Triticum turgidum L. durum (Desf.))
Source: PLoS One. 2018 Oct 23;13(10):e0206226. doi: 10.1371/journal.pone.0206226 (PMC6198983; doi:10.1371/journal.pone.0206226)
Supplement: S4 Table — KN, kernel number per spike. (DOCX) [file pone.0206226.s004.docx]

**S4 Table. Significant association between kernel number per spike** and **SNP markers in durum wheat.**

| Trait ^a^ | SNP markers | Chromosome bin | p | R^2^ |
| --- | --- | --- | --- | --- |
| KN | BE403597_2_A_1429 | 2BL2-0.36-0.50 | 0.0000 | 0.1482 |
| KN | BE426080_5_B_N_894 | C-5BL14-0.75* | 0.0000 | 0.1493 |
| KN | BE426413_6_A_Y_398 | C-6AL4-0.55 | 0.0000 | 0.1482 |
| KN | BE443930_1_A_N_94 | 1AL1-0.17-0.61 | 0.0000 | 0.1484 |
| KN | BE445506_7_B_Y_355 | 7BL10-0.78-1.00 | 0.0004 | 0.1287 |
| KN | BE489244_3_A_392 | 3AS4-0.45-1.00 | 0.0001 | 0.1488 |
| KN | BE494482_7_B_29 | 7B | 0.0001 | 0.1500 |
| KN | BE495786_1_B_108 | 1BL1-0.47-0.69 | 0.0000 | 0.1500 |
| KN | BE495896_5_B_276 | C-5BS4-0.43 | 0.0002 | 0.1190 |
| KN | BE497375_7_A_Y_191 | 7A | 0.0000 | 0.1246 |
| KN | BE499019_4_B_N_89 | 4BL5-0.86-1.00 | 0.0000 | 0.1373 |
| KN | BE500453_6_A_36 | 6AL4-0.55-0.90 | 0.0000 | 0.1486 |
| KN | BE517831_2_B_70 | C-2BL2-0.36 | 0.0007 | 0.1205 |
| KN | BE517877_2_A_95 | 2AS5-0.78-1.00 | 0.0009 | 0.0988 |
| KN | BE585744_7_A_Y_1417 | C-7AS8-0.45 | 0.0000 | 0.1483 |
| KN | BE586140_1_A_Y_77 | 1AS3-0.86-1.00 | 0.0001 | 0.1266 |
| KN | BE605063_1_A_37 | 1A | 0.0001 | 0.1447 |
| KN | BE607036_1_A_Y_334 | 1A | 0.0000 | 0.1483 |
| KN | BE607043_6_A_Y_163 | C-6AL4-0.55 | 0.0006 | 0.1223 |
| KN | BF201083_2_B_N_28 | 2B | 0.0000 | 0.1483 |
| KN | BF291549_1_B_N_577 | 1BS9-0.84-1.06 | 0.0009 | 0.0772 |
| KN | BF428726_1_A_Y_210 | 1A | 0.0000 | 0.1375 |
| KN | BF474493_4_A_N_40 | 4A | 0.0000 | 0.1558 |
| KN | BF482566_6_A_Y_285 | C-6AL8-0.90* | 0.0000 | 0.3237 |
| KN | BG607141_3_A_Y_180 | 3A | 0.0000 | 0.1483 |
| KN | BG607512_5_A_Y_411 | 5AL17-0.78-0.87 | 0.0001 | 0.1314 |
| KN | BM136727_6_B_631 | 6B | 0.0000 | 0.1507 |
| KN | BQ168329_2_A_Y_198 | 2A | 0.0007 | 0.1205 |
| KN | CD454152_5_A_Y_580 | 5AL17-0.78-0.87 | 0.0002 | 0.1385 |

^a^: KN, kernel number per spike.
