# Supplementary material for: Associations of canopy leaf traits with SNP markers in durum wheat (Triticum turgidum L. durum (Desf.))
Source: PLoS One. 2018 Oct 23;13(10):e0206226. doi: 10.1371/journal.pone.0206226 (PMC6198983; doi:10.1371/journal.pone.0206226)
Supplement: S6 Table — GNP, grain number per plant; GWP, grain weight per plant (g); RLMS, rachis internode length of main spike (cm); KGW, 1000-grain weight (g); SMS, number of spikelets on main spike; FW, fresh weight (g); NL, number of leaves; GRFW, growth rate of fresh weight; GRNL, growth rate for number of leaves; LA, leaf area (cm2); GRNR, growth rate for number of roots; GRLA, growth rate of leaf area. (DOCX) [file pone.0206226.s006.docx]

**S6 Table. SNP markers associated with multiple traits in durum wheat.**

| SNP loci | Trait type ^a^ | | |
| --- | --- | --- | --- |
|  | Leaf traits | Seedling traits | Agronomic traits |
| BE405834_1_B_Y_216 | FLL,SLL,TLL,FLW,SLW,FLA,SLA | FW,NL,GRFW,GRNL | GWP |
| BE405835_7_B_Y_166 | KGW | NL |  |
| BE406943_7_A_Y_313 | ACTL.TLCC | NL |  |
| BE425301_4_A_Y_160 | ACTL.TLCC |  | KGW |
| BE426214_6_A_N_191 | FLCC | FW,NR,LA,GRLA,GRFW,GRNR |  |
| BE438226_4_A_N_681 | FLCC | FW,NR,LA,GRLA,GRFW,GRNR |  |
| BE438495_6_B_Y_71 | FLCC | FW,NR,GRFW,GRNR |  |
| BE442666_4_B_Y_327 | FLL,SLL,TLL,FLW,SLW,FLA,SLA | NL,GRNL |  |
| BE442905_6_B_N_1225 | FLCC | FW,NR,LA,GRLA,GRFW,GRNR |  |
| BE443500_4_A_N_610 | FLCC | FW,NR,GRFW,GRNR |  |
| BE443538_5_A_1436 | FLL,SLL,TLL,FLW,SLW,TLW,FLA,SLA,TLA,TATL | FW,NL,GRFW,GRNL | GNP,GWP,RLMS |
| BE443540_7_B_N_1397 | FLL,SLL,TLL,FLW,SLW,TLW,FLA,SLA,TLA,TATL | FW,NL,GRFW,GRNL, |  |
| BE443973_4_A_105 | ACTC,TLCC | GRNR |  |
| BE444144_2_B_92 | SLCC |  | SMS |
| BE444644_5_A_Y_287 | ACTC,TLCC | GRNR |  |
| BE444858_4_A_Y_87 | ACTC,TLCC | GRNR |  |
| BE445587_7_A_N_347 | FLL,SLL,TLL,FLW,SLW,FLA,SLA,ACTL,TLCC | FW,NL,GRFW,GRNL,GRNR |  |
| BE490763_2_A_1462 | FLCC | FW,NR,LA,GRLA,GRFW,GRNR | GNP,GWP |
| BE494023_4_A_N_380 | FLCC | FW,NR,LA,GRLA,GRFW,GRNR |  |
| BE494028_7_A_Y_108 | FLCC | FW,NR,GRFW,GRNR |  |
| BE517711_5_B_49 | FLCC | FW,NR,LA,GRLA,GRFW,GRNR |  |
| BE517914_3_A_Y_81 | FLL,SLL,TLL,FLW,SLW,TLW,FLA,SLA,TLA,TATL | GRRL |  |
| BE590521_6_B_N_331 | FLL.SLL,TLL,FLA,SLA,TLA,TATL | FW,NR,LA,GRLA,GRFW,GRNR | GNP,GWP,RLMS |
| BE591423_5_B_Y_580 | FLCC | FW,NR,LA,GRLA,GRFW,GRNR |  |
| BE591861_4_A_Y_848 | ACTL,TLCC | GRNR |  |
| BE606541_6_B_Y_676 | FLL,SLL,TLL,FLW,SLW,FLA,SLA | FW,NL,GRFW,GRNL | GWP |
| BF292614_6_B_189 | FLCC | FW,NR,GRFW,GRNR |  |
| BF145580_2_A_107 | FLL,SLL,TLL,FLW,SLW,FLA,SLA | NL, GRNL |  |
| BF201102_5_A_Y_154 | ACTL,TLCC | GRNR |  |
| BF202975_2_B_280 | ACTL,TLCC | GRNR |  |
| BF292414_1_A_78 | FLCC | FW,GRFW |  |
| BF474552_7_B_Y_127 | ACTL,TLCC | GRNR |  |
| BF474569_1_A_Y_382 | FLCC | FW,NL,GRFW,GRNL, |  |
| BF478690_1_B_Y_78 | ACTL,TLCC | GRNR |  |
| BF482529_7_A_304 | ACTL,TLCC | GRNR |  |
| BF484496_1_B_N_150 | FLCC | FW,NR,LA,GRLA,GRFW,GRNR |  |
| BF484606_1_A_390 | FLCC | FW,NR,LA,GRLA,GRFW,GRNR |  |
| BF485305_1_A_Y_29 | FLCC | FW, NR,GRFW,GRNR |  |
| BG262734_3_A_N_190 | FLCC |  | GNP |
| BG263233_1_B_825 | ACTL,TLCC | NL |  |
| BG274119_1_A_Y_221 | FLCC | FW, NR, GRFW,GRNR |  |
| BG313767_1_B_107 | ACTL,TLCC | GRNR |  |
| BG314205_1_B_33 | FLL,SLL,TLL,FLW,SLW,TLW,FLA,SLA,TLA,TATL | FW,NL,GRFW,GRNL | GNP,GWP,RLMS |
| BG604857_7_B_N_74 | FLCC | FW,NR,GRFW,GRNR |  |
| BG607308_5_A_Y_101 | ACTL,TLCC | GRNR |  |
| BI479113_5_B_534 | FLCC | FW,NR,GRFW,GRNR |  |
| BM137384_5_A_444 | FLCC | FW,NR,LA,GRLA,GRFW,GRNR |  |
| BQ159615_6_B_N_189 | FLCC | FW,NR,GRFW,GRNR |  |
| BQ167580_3_A_Y_342 | FLCC | NL |  |
| BQ169448_6_B_252 | FLCC | FW,NR,LA,GRLA,GRFW,GRNR | LMS |
| BQ169669_7_A_Y_378 | ACTL,TLCC | GRNR |  |
| BQ172173_2_B_Y_157 | ACTL,TLCC | GRNR,NL |  |
| CD452629_7_B_58 | AVTL,TLCC | GRNR |  |
| CD453593_6_A_N_238 | FLCC | FW,NR,LA,GRLA,GRFW,GRNR |  |
| BE405835_7_B_Y_166 | KGW | NL |  |
| BE586140_1_A_Y_220 | KGW |  | GNP |

^a^ GNP, grain number per plant; GWP, grain weight per plant (g); RLMS, rachis internode length of main spike (cm); KGW, 1000-grain weight (g); SMS, number of spikelets on main spike; FW, fresh weight (g); NL, number of leaves; GRFW, growth rate of fresh weight; GRNL, growth rate for number of leaves; LA, leaf area (cm^2^); GRNR, growth rate for number of roots; GRLA, growth rate of leaf area.
